# African Pharmacogenomics Consortium: Consolidating pharmacogenomics knowledge, capacity development and translation in Africa

**DOI:** 10.12688/aasopenres.12965.1

**Published:** 2019-06-04

**Authors:** Collet Dandara, Collen Masimirembwa, Yosr Z. Haffani, Bernhards Ogutu, Jenniffer Mabuka, Eleni Aklillu, Oluseye Bolaji, H3Africa

**Affiliations:** 1 Pathology & Institute of Infectious Diseases and Molecular Medicine, University of Cape Town, Cape Town, 7925, South Africa; 2 African Institute of Biomedical Science and Technology, Harare, Zimbabwe; 3 Higher Institute of Biotechnology Sidi Thabet, Manouba University, Ariana, LR17ES03, Tunisia; 4 Centre for Research in Therapeutic Sciences, Strathmore University, Nairobi, Kenya; 5 Secretariat, The African Academy of Sciences (AAS), Nairobi, Kenya; 6 Department of Laboratory Medicine, Karolinska Institutet, Stockholm, Sweden; 7 Department of Pharmaceutical Chemistry, Obafemi Awolowo University, Ile-Ife, Nigeria

**Keywords:** pharmacogenomics, pharmacogenetics, Africa, adverse drug response (ADR), genotype, phenotype

## Abstract

The African Pharmacogenomics Consortium (APC) was formally launched on the 6th September 2018. This white paper outlines its vision, and objectives towards addressing challenges of conducting and applying pharmacogenomics in Africa and identifies opportunities for advancement of individualized drugs use on the continent.  Africa, especially south of the Sahara, is beset with a huge burden of infectious diseases with much co-morbidity whose multiplicity and intersection are major challenges in achieving the sustainable development goals (SDG), SDG3, on health and wellness. The profile of drugs commonly used in African populations lead to a different spectrum of adverse drug reactions (ADRs) when compared to other parts of the world. Coupled with the genetic diversity among Africans, the APC is established to promote pharmacogenomics research and its clinical implementation for safe and effective use of medicine in the continent.  Variation in the way patients respond to treatment is mainly due to differences in activity of enzymes and transporters involved in pathways associated with each drug’s disposition.  Knowledge of pharmacogenomics, therefore, helps in identifying genetic variants in these proteins and their functional effects. Africa needs to consolidate its pharmacogenomics expertise and technological platforms to bring pharmacogenomics to use.

## Disclaimer

The views expressed in this article are those of the authors. Publication in AAS Open Research does not imply endorsement by the AAS.

## The problem to be addressed by the African pharmacogenomics consortium

Traditionally, disease patterns are characterised with infectious diseases (malaria, TB, HIV, cholera, neglected tropical diseases) being the major cause of morbidity and mortality in developing countries in Africa, Asia and South America ( Srivastava *et al*., 2018). On the other hand, non-communicable diseases such as cancer, cardiovascular disease, and neuropsychiatric disorders have been associated with developed countries of Europe, North America and Japan ( Guthold *et al*., 2018). However, changes in life style in developing countries have resulted in what is termed the ‘epidemiological transition’ where these countries now bear the double burden of infectious and non-communicable diseases ( Juma *et al*., 2018; Keates *et al*., 2017). This has increased the disease burden in these countries where Africa, which has 10% of the world population, now carries 25% of the global disease burden (See AfricaRenewal, 2016–2017; Crisp, 2011). This has in turn increased the need for treatment interventions to reduce morbidity and mortality. Whilst the use of medicines has been associated with huge reductions in mortality thereby increasing life expectancy, some medicines such as anti-retroviral drugs (ARVs) have been associated with a huge surge in adverse drug reactions (ADRs) where up to 80% of ADRs in some sub-Saharan Africa are now due to ARVs ( Ampadu *et al*., 2016; Appiah, 2012; Nemaura *et al*., 2012; Rajman *et al*., 2017; Sarfo *et al.*, 2014a). On the other hand, efforts to combat non-communicable disease have shown a widespread lack of efficacy of some medicines used in treating hypertension ( Fontana *et al*., 2014) and breast cancer ( Li *et al*., 2017). The burden of ADRs and poor efficacy translates to disability, death and huge costs to the already constrained healthcare systems of Africa. It is this burden of poor safety and lack of efficacy of medicines in African populations that the African Pharmacogenomics Consortium seeks to address. This will be done by quantifying the disease burden, understanding the underlaying biomedical mechanisms, evaluating costs to the healthcare systems and finding interventions for improved treatment outcomes using a responsible innovation (RI) approach.

ADRs are unwanted drug effects and have considerable economic as well as clinical costs as they often lead to hospital admissions and prolongation of hospital stay which increases pressure on health care systems that are often overstretched ( Sultana *et al*., 2013). Estimates from USA and Canada show that ADRs account for 4–30% and 6–35% hospital admissions and hospitalization, respectively, while France reports at least, 100,000 patients presenting with ADRs per annum. The Food and Drug Administration (FDA) of the United States of America reports 58,000–106,000 annual deaths due to ADRs ( Sultana *et al*., 2013). ADRs add to the healthcare cost as illustrated by Watanabe *et al*. (2018) in a study where they report on an estimated cost of prescription drug-related morbidity and mortality resulting from non-optimal medication therapy of at least $500 billion for 2016. This is equivalent to nearly 15% of total US healthcare expenditure and way above most GDPs in African countries. Another study from the United Kingdom, reported that ADRs increased the mean hospital stay from an average of 8 days in patients without ADRs to 20 days in patients with ADRs ( Davies *et al*., 2009) which was accompanied by an increased risk of mortality in patients who experienced ADRs.

Through global coordinated efforts, medicine supply including new drugs to treat poverty related diseases is increasing but this effort is not matched well with local capacity to monitor patient safety in indigenous African populations. The impact of the burden of ADRs in Africa with respect to people affected, drugs involved and cost to the healthcare system is poorly characterized. Available data on ADRs in Africa is scarce except for a few studies from Kenya ( Aminkeng *et al*., 2014), Ethiopia ( Petros *et al*., 2017a; Yimer *et al*., 2012), Ghana ( Sarfo *et al*., 2014), South Africa ( Aminkeng *et al*., 2014), Zimbabwe ( Nemaura *et al*., 2012) and in a few other African countries of which most are single hospital studies. This is reflected by low participation in pharmacovigilance programs where, by 2016, only 35 countries were participating in the WHO Program for International Drug Monitoring (PIDM) which involves reporting of individual safety case report (ICSR). Africa contributes a mere 0.88% ICSR to this VigiBase ^R^, with South Africa being the most active ( Ampadu *et al*., 2016). Despite this low reporting for many drugs, data shows that ADRs from ARVs and some antibiotics are 5–10% higher in Africans compared to the rest of the world ( Ampadu *et al*., 2016). Whereas, in most developed countries, ADRs have also been characterized ( *e.g*., for drugs such as Nonsteroidal anti-inflammatory drugs (NSAIDs), coumarins, antibiotics, anticancer, and beta-blockers), facilitating their recognition and prevention; ADRs in African populations are mainly on the backbone of antiretroviral ( Ampadu *et al*., 2016; Mouton *et al*., 2016; Rajman *et al*., 2017) accounting for at least 30% of ICSRs, followed by anti-tuberculosis and antimalarial therapy, respectively ( Ampadu *et al*., 2016; Birbal *et al*., 2016; Mouton *et al*., 2016).

To our knowledge, there is no published data on the burden of ADRs with respect to mortality at national or regional level in Africa, there are very few studies that have evaluated the economic impact of ADRs. A recent study conducted by Management Sciences for Health, a Virginia–based international nonprofit organization, showed that 6.3% of hospital admissions in Sub-Saharan Africa were direct consequences of an ADRs, while between 6.3% and 49.5% of hospitalized patients developed ADRs ( Appiah, 2012). A study in South Africa showed that 1 in 12 admissions was because of an ADR, and that ADRs were associated with drugs mostly used for the treatment of HIV and TB ( Mouton *et al*., 2016). There is also a distinct complex disease-disease, and drug-disease as well as drug-drug interaction profiles emerging in sub-Saharan Africa where HIV patients have been shown to have a high risk for cardiovascular diseases ( Keates *et al*., 2017) and where some ARVs have been shown to increase the risk for metabolic disorders in these patients ( Keates *et al*., 2017). For example, at least 40% of HIV/AIDS patients on combination antiretroviral therapy (cART) in South Africa present with hypertension ( Nlooto, 2017). Most drugs used for the treatment of non-communicable diseases were developed after clinical trials carried out in Caucasian and Asian populations with a poor or no representation of African populations, except in trials on HIV/AIDS ( GBD 2016 and HALE collaborators, 2017; Kharsany & Karim, 2016). This has led to reports of ADRs in African patients with drugs that sometimes have not shown any such effects in Caucasian populations ( Taylor, 2018). Moreover, some drugs that have proven efficacious in Caucasian populations have not shown similar action in African populations ( Fontana *et al*., 2014; Li *et al*., 2017). In particular, the massive use of cART for HIV/AIDS has led to many people living with HIV for longer periods of time, allowing ADRs associated with long term cART use to manifest ( Ghosn *et al*., 2018; Kharsany *et al*., 2018; Montjane *et al*., 2018; Soko *et al*., 2018). A distinct population specific drug interaction profile between rifampicin and efavirenz in black African and Caucasian populations, has necessitated different efavirenz dose modification strategies during rifampicin co-treatment ( Habtewold *et al*., 2015; Habtewold *et al*., 2017). The impact of rifampicin enzyme induction in reducing efavirenz plasma exposure observed in Caucasian or Asians was not replicated in black Africans, partly due to pharmacogenetic variations ( Mukonzo *et al*., 2014a; Ngaimisi *et al*., 2011). Recent studies recommended pharmacogenetic-based EFV dose modification during rifampicin based anti-tuberculosis co-treatment for sub Saharan African population ( Mukonzo *et al*., 2016; Mukonzo *et al*., 2014b).

The underlying mechanisms of high frequency of ADRs and poor efficacy of some medicines in African populations remain largely unknown. Studies in European populations have shown that most ADRs are concentration dependent. A high concentration of the parent drug and/or its metabolites can result in exaggerated primary pharmacological effects and/or appearance of new and undesirable secondary pharmacological effects. The high concentrations could be due to the physicians’ deliberate effort to increase therapeutic effect or errors in prescription. A large percentage of ADRs due to high drug exposures have been attributed to reduced metabolic activity of enzymes responsible for the metabolism and excretion of the drug of interest. For instance, the CYP3A enzyme activity is significantly lower in Tanzanians than Swedes or Koreans ( Diczfalusy *et al*., 2008; Mirghani *et al*., 2006). Factors that affect drug metabolism and disposition (drug metabolising enzymes and transporters) have therefore been extensively studied as the mechanism behind most observed ADRs. Two major mechanisms have been demonstrated to be responsible for variable drug exposures; enzyme or transport inhibition or induction, and genetic variation in genes coding for drug metabolising enzymes or drug transporters associated with reduced or increased function. A study in about a thousand patients showed that interactions associated with risk for ADRs involved 50% due to drug-drug interactions, 34% drug-gene interactions and 19% of drug-drug-gene interactions ( Verbeurgt *et al*., 2014).

The possible contribution of these mechanism to the ADRs observed in African populations are poorly understood due to several reasons including, lack of knowledge on the extent of pharmacogenetic variation in African populations ( Rajman *et al*., 2017), lack of clinical pharmacogenetic studies to evaluate the role of the known genetic variants in observed ADRs, and lack of known enzymes and transporters involved in the disposition of many drugs commonly used in African populations such as anti-parasitic drugs. There is therefore a great need to investigate the role of drug-drug, drug-gene and drug-drug-gene interactions as risk factors for ADRs in African populations. The African Pharmacogenomics Consortium (APC) has therefore identified genomic factors as important factor in understanding ADRs in African populations and intends to come up with interventions for improved treatment outcome. In a contribution to domestication of precision medicine, the consortium will foster development of robust electronic health records for patients and decision support systems to translate, share and communicate pharmacogenomics results to healthcare providers and patients, and to provide evidence-based recommendation for policy makers to revise treatment guidelines relevant for African populations.

## Pharmacogenomics as the solution

Pharmacogenomics utilizes a person’s genome (or genetic makeup), to identify drugs and drug doses that are likely to work best for that particular person, or drugs that are likely to cause ADRs. In Africa, there have been several initiatives filling the gaps that will eventually inform new ways of improving health, two of these include MalariaGen and H3Africa (see Table 1). However, the focus of most of these initiatives has been primarily on the genomics of disease susceptibility with little or no pharmacogenomics. African health care systems are complex, involving contemporary and herbal medicines. Thus, pharmacogenomics could enable a better understanding of the basis of both western and traditional medicine leading to better integration ( Thomford *et al*., 2018; Xin *et al*., 2019).

**Table 1.** A list of some of the common genomics initiatives in Africa.

INITIATIVE	FOCUS	ADDRESS/ CONTACT
African Pharmacogenomics Consortium (APC)	The genetics of drug effectiveness (meetings, training workshops, conferences, collaborations)	Current initiative (website to be developed) ( bsiddondo@strathmore.edu)
The African Society for Human Genetics (AfSHG)	Annual conferences/meetings	https://www.afshg.org/
African Human Genome Initiative	Lectures, conferences, discussions	www.africagenome.co.za
H3Africa	Genomics and environmental determinants of disease	https://h3africa.org
MalariaGEN	Malaria genomic epidemiology Network, focussing on effects of genetic variation on the biology and epidemiology of malaria	www.malariagen.net
H3ABioNet	pan-African bioinformatics network	https://www.h3abionet.org/
the Southern African Human Genome Project	Understanding of DNA variation among southern Africans and how this impact on the health of the people of our country.	https://sahgp.sanbi.ac.za
African Genome Variation Project	Aims to collect essential information about the structure of African genomes to provide a basic framework for genetic disease studies in Africa	https://www.sanger.ac.uk /science/collaboration/ african-genome-variation-project

## Pharmacogenomics in drugs and diagnostics discovery, development and deployment

The two most important concerns for new drug development are efficacy and safety. Generally, the process of drug discovery starts with the identification of a potential target at which the drug can act. The target can be an enzyme in a vital pathway, a receptor, a transporter, a protein in signal transduction or any protein important in disease manifestation. Currently, about 300 targets of the potentially 5000 drug targets are being exploited for drug discovery. These are mostly proteins (e.g. enzymes and receptors) that are coded for by genes that exhibit genetic polymorphisms. Knowledge of pharmacogenomics at this level has helped in the development of anticancer drugs that work in patients of specific genotypes and thus informed the development of companion diagnostic tools to identify such responders in the clinical setting (see Pharmacogenomics Knowledge Database).

The pharmaceutical industry has reported that up to 60% of compounds in their discovery and development pipelines have a pharmacogenomics component ( Zhang *et al*., 2012) necessitating the need of a pharmacogenomic strategy in the whole discovery and development value chain. There is an Industry Pharmacogenetics working group that provides the relevant strategic input on this matter for its membership. Genetic studies in conjunction with gene expression, proteomic, and metabonomic analyses provide a powerful tool to identify molecular subtypes of disease. Using these molecular data, pharmacogenomics has the potential to impact on the drug discovery and development process at many stages of the pipeline, contributing to both target identification and increased confidence in the therapeutic rationale.

In the drug discovery and development value chain, pharmacogenomics can be useful at the following stages:

(1) *Drug target identification and validation–* characterising the heterogeneity of drug targets and variable target-chemical interactions with potential pharmacodynamic effects. This can result in avoiding certain drug targets or developing a companion diagnostics strategy that will be used to identify responder and non-responder patient subgroups in the clinical setting. Genetic variation in the human CD4 cells receptor, CCR5 inspired the discovery of the cells entry inhibitor, maraviroc ( Dorr *et al*., 2005; Perry, 2010; Veljkovic *et al*., 2015) and a companion diagnostic for its use in patients likely to benefit from the drug ( Kim *et al*., 2016; Whitcomb *et al*., 2007). Pharmacogenomics has already been used in oncology to demonstrate that molecular data facilitates assessment of disease heterogeneity, and thus identification of molecular markers of response to drugs such as imatinib mesylate (Gleevec) and trastuzumab (Herceptin). Knowledge of genetic variation in a target allows early assessment of the clinical significance of polymorphism through the appropriate design of preclinical studies.

(2) *Lead and candidate drug discovery phase* – *in vitro* characterisation of compounds for metabolism or transport by proteins that exhibit functionally important variations. This will result in either molecular design to avoid compounds likely to have unfavourable pharmacokinetics and pharmacodynamics in some patient groups or to design phase I clinical studies that target affected enzymes or transporters. In lead and candidate drug discovery, assessment of drug metabolising enzyme and drug transporters pharmacogenetics studies are performed to inform selection of suitable candidates for first time in man and the subsequent design of clinical trials ( Raymer & Bhattacharya, 2018)

(3) *Phase I and II clinical trials* – In clinical studies, pharmacogenetic tests are used for stratification of patients based on their genotype, which corresponds to their metabolizing capacity. This prevents the occurrence of severe ADRs and helps in providing better outcomes from clinical trials. This can also reduce attrition of drug compounds.

(4) *Phase III* – identification and validation of the function of common genetic variants on drug PK and PD, design of preventive trials based on predisposed PGx biomarkers, development of dosage algorithm based on PGx and discovery of ADRs related PGX biomarkers.

(5) *Phase IV clinical trials–* identification and validation of the function of rare genetic variants on drug PK, PD and ADRs, validation of the PGx biomarkers related to ADRs and design of prospective study in prevention of ADRs based on PGx biomarkers ( Wen *et al*., 2015). In this regard members of the APC have conducted clinical pharmacogenetic studies on the use of efavirenz in HIV patients ( Dhoro *et al*., 2015; Habtewold *et al*., 2015; Nemaura *et al*., 2012; Ngaimisi *et al*., 2011; Nyakutira *et al*., 2008; Olagunju *et al*., 2015a; Olagunju *et al.*, 2015b; Swart *et al.*, 2013), antiretroviral and antimalarial drug interactions ( Maganda *et al*., 2016; Mutagonda *et al*., 2017), genetic biomarkers for antiretroviral and anti-tuberculosis drug induced hepatotoxicity ( Petros *et al*., 2017a; Petros *et al*., 2017b; Petros *et al*., 2016), imatinib in the treatment of chronic myelogovenous leukaemia ( Adeagbo *et al*., 2016), and the pharmacokinetics of rosuvastatin in African populations ( Soko *et al*., 2016; Soko *et al*., 2018) and showed the potential importance of pharmacogenetic biomarkers in the optimal use of these drugs in African populations.

If the emerging genomic diversity of African populations is also observed in clinically significant pharmacogenes, that diversity will therefore present an opportunity for Africa to actively participate in the drug discovery and development process. This can be done through several ways including (i) opportunities to discover disease receptor subtypes that can help provide proof of concept through validation of the selected target as suitable for drug discovery, (ii) having higher frequency of important PGx variants not commonly found in other world populations, thus making it strategically and economically attractive to conduct phase I clinical studies in African populations, and (iii) biomarker discovery for ADRs will be more productive in a population that shows a wide genetic diversity of involved gene(s). Africa and the rest of the world is currently not taking full advantage of this opportunity despite leading world scientists in the field such as Rotimi (See Newsweek interview) and Tishkoff (See Scientifc American blog) highlighting the perils of excluding African genomics in the advancement of medical research. The APC will therefore build a case for the exploitation of this opportunity through engaging biopharmaceutical companies and biotechnology companies for joint ventures in drugs and diagnostics discovery and innovation.

## Vision of African pharmacogenomics consortium

The vision of the APC is to explore the diverse African genome for better health in the continent. The consortium aims to characterise the genomes of African populations to unravel crucial pharmacogenes for the improvement of quality of life of African patients. This vision will be achieved through consolidation of pharmacogenomics research and its implementation in Africa through strategic collaborations of Africans based in Africa leveraging expertise from international partners.

## Historical perspective on APC

The vision of the consortium is built through multiple functional interactions and partnership of the network members which is supported by a strong history. Formation of the APC can be traced to August 2003, when African scientific experts focussing on pharmacogenomics met in Nairobi, Kenya, with the aim of strengthening pharmacogenomics research in Africa, through collaborations and postgraduate students training. The need of this collaboration was raised following the incorporation of some pharmacogenomic tests and clinical decision making, developed on Caucasian and Asian populations, which have proved not to be fully transferable to African populations through algorithms because of the extent of genetic diversity in these populations. Thus, pharmacogenomic characterisation of African populations needs to be carried out as such knowledge has the potential to save lives and reduce healthcare costs through reduction in hospital admissions, mortality thereby freeing resources for use in other healthcare areas. Adoption of pharmacogenomics in Africans can, thus, lead to improved drug effectiveness, and prevent morbidity and mortality ( Ashley *et al*., 2010; Mallal *et al*., 2008; Squassina *et al*., 2010).

## Objectives of African pharmacogenomics consortium

### A. Awareness of pharmacogenomics among Africans

APC will create awareness in pharmacogenomics through training by offering short courses and degree programmes in partnership with accredited universities. In addition, dissemination of pharmacogenomics knowledge will form part of awareness and this will be achieved through publications (policy briefs, opeds, etc). The consortium will organise workshops and demonstrations to train stakeholders on the use of the delivered technologies regarding pharmacogenomics. Special emphasis will be conducted on “train the trainer” outreach so that the information will be disseminated to the greatest extent possible. It will coordinate and manage publications of the project findings in pharmacogenomics, biological and medicinal trade magazines and scientific journals. It will also establish an online consultation platform ’Consult Expert’, implement, manage, maintain and further grow databases of contacts and links that can be used by the consortium to specifically target messages to stakeholder’s groups and actors (hospitals, clinics, schools, national educational authorities, training centres, SMEs, associations, social media and forums and others). Lastly, APC will carry out public engagements for pharmacogenomics through the media (print, digital, audio visual), publish scientific knowledge into popular messages, including multi-lingual concepts targeting the different languages in Africa, and also develop a non-verbal communication tool based on symbols.

### B. Research and training on pharmacogenomics in Africa

APC will work towards building integrated capacities for pharmacogenomics in terms of bioanalysis, bioinformatic, clinical trials and biobanking/ genomic analysis. This will enable African researchers to generate relevant research questions which they have capacity to answer. As far as world trends are concerned, Africa’s current contribution is insignificant ( Adedokun *et al*., 2016), yet the continent is a “gold-mine” with respect to the wide genetic diversity of the human genome as well as its co-evolution with some of the problematic pathogens such as tuberculosis bacteria, which could provide answers to some of the currently elusive genetic markers of susceptibility, response and co-evolution. Some of the major reasons for this low research capacity are poor infrastructure for research at public research institutions such as universities, and lack of a research and innovation-based biopharmaceutical and biotechnology industry to invest in genomic research. This has also meant that the few skilled genomics scientists have been trained abroad as there is no local capacity for such training. Governments and the private sector in Africa need to invest in infrastructure, technology and skilled manpower to enable Africa to participate in the genomics driven development in life sciences.

### C. Implementation of pharmacogenomics in Africa

In translating African pharmacogenomics knowledge, optimization of available pharmaceuticals is a major priority as these drugs are already in use. The conduct of bridging studies is, thus, most relevant in African populations. This is supported by observations in China and Japan for drugs in which their populations have not been part of during clinical trials, are not allowed for use in their populations without first carrying out relevant bridging studies. The next challenge in improving human health is being tackled through precision medicine, thus, APC seeks to ensure domestication of precision medicine in the African health system. African populations are unique in that they use a diverse health care system; thus, APC seeks to target health system strengthening of medicinal products use (traditional and conventional). Coding and sharing of best practices in African pharmacogenomics will be at the core of its implementation strategies. In order to support the health care system, APC will develop and regularly update pharmacogenomics implementation guidelines for African populations and these should benefit from seamless link with the pharmacovigilance and clinical trials platforms in Africa. APC will harness the genomic diversity Africans in drugs and diagnostics discovery/commercialisation in partnership with local and international biotechnology and biopharmaceutical companies. To increase uptake of pharmacogenomics, APC will partner in the development of curricula for training in pharmacogenomics. To retain and equip practitioners of pharmacogenomics APC will create regional hubs of excellence in pharmacogenomics. The consortium will regularly develop matrices/models for pharmacogenomics implementation impact assessment. It seeks to be the “African voice” on pharmacogenomics and affiliate with appropriate international bodies including but not limited to genomic societies.

## Recommendations by the African pharmacogenomics consortium/network (APC)

### (i) Capacity development for pharmacogenomics in Africa

APC aims to develop research leadership impactful of research on Africa and led by Africans. Currently most research in genomics is led or coordinated by researchers in Europe or America in which African researchers have acted as sample collectors ( Dandara *et al.*, 2014; H3Africa sustainability). It is, therefore, not surprising to come across genomics research on Africans published without acknowledgement of African authors, and in the few cases where African researchers are involved they are ‘middle-of-the-pack’ insignificant co-authors. Although Africa has seen some leap in the development of human capital resources for genomics research, there has not been much focus on pharmacogenomics. It is our intention that APC should develop an infrastructure and programs that support harmonisation of participant recruitment and phenotype recording. There are very few centres in Africa that are equipped for pharmacogenomics phenotype analysis as well as genome characterisations. This will be associated with the establishment of biobanks/biorepositories to support pharmacogenomics research and linked to local capacity for laboratory drug and genomic analysis. We would like to strengthen these centres and make them core-facilities where students and researchers can get access on a short-term basis to resolve issues/challenges they would be facing in their research at any particular moment, through training and analysis of their samples.

### (ii) Education/training support and ethical, legal, and social issues (ELSi)

APC seeks to take stock of the number of researchers working on pharmacogenomics in Africa, increase this number with training of MSc/PhD graduates and incorporating ethical, legal and social issues (ELSi) that are sensitive to African populations. This will reduce cases of ethics dumping. Currently, alignment of ELSi on African genomics is led by researchers from outside Africa, as can be viewed through published literature. While acknowledging the Western view on ethics, it is our view that, the African voice should find space and lead in the discourse, if we are going to have ethics that respond to African values. Moreover, the continent has varied local ethics regulations which require harmonisation for across country initiatives such as the APC. This could be achieved through influencing policy at the level of continental institutions/bodies such as the African Union Development Agency (AUDA), a technical arm of the African Union (AU).

There are no programs that capture pharmacogenomics in African universities, thus, there is a need to develop innovative courses for training MSc/PhD students in these universities, leveraging expertise from APC hubs of excellence, and APC network of experts. In addition, the APC would endeavour to carry out community engagements by domesticating pharmacogenomics through presentation of the topics and issues in the context of people’s social and cultural experiences. This will include qualitative engagements on safety and efficacy of medicines through focus-group discussions and interviews. Members in the APC will leverage their rich history of training students across Africa to accomplish this task. It is expected that this initiative should further empower such trained individuals to compete for grant funding thereby putting into use knowledge acquired. APC will build on existing platforms to leverage on their support and endeavour that projects running under its banner meet the ethical, legal, and socially appropriate standards for research. APC will also seek the harmonisation of participant recruitment and engagements for pharmacogenomics research and implementation in Africa.

### (iii) Resource development and utilization

APC will work towards building integrated capacities for pharmacogenomics. African entities such as New Partnership for Africa’s Development (NEPAD) and the African Academy of Sciences (AAS) could be used as sounding boards for across the board implementation, resource mobilisation and utilization. APC will work for recognition from WHO, which is respected by African governments, making it easier for adoption of its recommendations. It is noteworthy that the WHO developed a position paper on pharmacogenomics (WHO Drug Information Vol 19. No. 1, 2005). Though now old, it is aligned to the now well-developed guidelines for pharmacogenomics by European Medicines Agency (EMA) (EMA February, 2018) and a series of pharmacogenomics guidelines by the FDA and by industry working group on pharmacogenomics ( Patterson *et al*., 2011). It is thus imperative that the APC spearheads the development of a position on pharmacogenomics for Africa.

### (iv) Database for clinical pharmacogenomics implementation guidelines for African populations

 The biggest resource that African populations have is the genomic diversity. This diversity probably holds the keys to unlocking the identification of genomic determinants of susceptibility to complex diseases such as diabetes and determinants of differential response to drug treatments. However, for the effective use of African genomes, baseline frequencies of pharmacogene variants need to be developed. After pharmacokinetic and pharmacodynamic studies, the APC should be in a position to come up with recommendations for priority pharmacogenomics for different drug/disease combinations in African patients. APC will lead the developing and updating of recommendations for implementation of pharmacogenomics in African populations.

### (v) Building sustainable governance in pharmacogenomics in Africa

The consortium will aim to put into place ethical and sustainable structures in the area of pharmacogenomics research with respect to sample/data collection and storage, data sharing and release, and student training exchange. This will be achieved through structured governance. For any project that the consortium will embark on, a principal applicant (project coordinator) and co-applicants will be chosen from participating countries to form a steering committee (SC) as the decision-making organ. The SC will provide general direction and scientific guidance to the proposed work. The project coordinator will act as the communications liaison person for such an application and will play a coordinating role for all the proposed research activities.

## Conclusions

The WHO urged the implementation of pharmacovigilance centres in Africa to raise the awareness of ADRs (US Agency for International Development). A recent report on the action taken regarding regulatory authorities in African nations showed that it “requires the necessary infrastructure and resources including laws, systems and structures, human resources (in terms of numbers, knowledge and skills) and financial resources to execute their mandate” including pharmacovigilance to monitor drug safety (see report from the Africa Pharmacovigilance Meeting 2012). In this, the APC will be implementing hubs of excellence in African countries to promote pharmacogenomics and pharmacovigilance according to the regional needs of the continent. Interestingly, the APC support the wise words of the South African revolutionary, political leader, and philanthropist Nelson Mandela, ‘We must face the matter squarely, that where there is something wrong in how we govern ourselves, it must be said that the fault is not in the stars, but in ourselves. We know that we have it in ourselves as Africans to change all this. We must assert our will to do so; we must say there is no obstacle (large) enough to stop us bringing about an African renaissance’ ^1^ ( Herbert & Gruzd, 2017).

## Data availability

### Underlying data

No data are associated with this article

## Footnotes

^1^ Mandela N, Statement of the President of the Republic of South Africa, at the Organization of African Unity (OAU) Meeting of Heads of State and Government, Tunis, Tunisia, 13 June 1994.
