# Supplementary material for: African Pharmacogenomics Consortium: Consolidating pharmacogenomics knowledge, capacity development and translation in Africa: Consolidating pharmacogenomics knowledge, capacity development and translation in Africa
Source: AAS Open Res. 2019 Jun 4;2:19. [Version 1] doi: 10.12688/aasopenres.12965.1 (PMC7194139; doi:10.12688/aasopenres.12965.1)
Supplement: Peer review history

# Peer review: Reviewer response for version 1

**DOI:** 10.21956/aasopenres.14044.r27031

**Published:** 2019-07-19

**Authors:** Ann K. Daly

**Affiliations:** 1 Institute of Cellular Medicine, Medical School, Newcastle University, Newcastle upon Tyne, UK

This is a good overview of pharmacogenomics research in Africa which focusses well on the specific challenges encountered in clinical implementation on the continent. I agree that establishing the nature of genetic variation in the various genes across the diverse African populations is the main priority. I recommend that the authors should encourage using a broad definition of the term pharmacogenomics as it is often difficult to distinguish between genomics more generally and pharmacogenomics specificially. Possibly pharmacogenomics and "related areas of genomics" is a good way to go.

I confirm that I have read this submission and believe that I have an appropriate level of expertise to confirm that it is of an acceptable scientific standard.

# Peer review: Reviewer response for version 1

**DOI:** 10.21956/aasopenres.14044.r27002

**Published:** 2019-07-01

**Authors:** Ahmed Rebai

**Affiliations:** 1 Centre of Biotechnology of Sfax, University of Sfax, Sfax, Tunisia

The paper addresses the objectives and challenges of the newborn African Pharmacagenomics Consortium (APC).

 The APC has been created few months ago by scientists from five African countries under the H3Africa umbrella and with the coordination of the African Academy of Science. It is a very welcome initiative to coordinate and stimulate African efforts for the study of genomics components involved in drug metabolism and mainly adverse drug reactions (ADR). The consortium, when reaching a good maturity level, will be a platform for regulated data sharing and collaborative research in the field of pharmacogenomics, where data on African populations are still scarce and dispersed.

 The paper provides a good description of the current and future challenges in the pharmacogenomics field worldwide, and in Africa. It then gives a set of recommendations to foster development of capacities, resources and sustainable governance of research structures and networks in pharmacogenomics within the continent. 

 The paper is very well written with clear ideas and objectives. However, I think that the description of current African capacities and data which are available at country level in publications or national initiatives are not well covered. A literature search with the appropriate keywords and country affiliation would allow access to such data. I recommend the authors to address this issue in order to gain visibility on the existing capacities in Africa and widen their consortium. One of the corner stones in strengthening and widening the consortium will be collect such data in a database and make it publicly available through the African Academy of Science platform.

I confirm that I have read this submission and believe that I have an appropriate level of expertise to confirm that it is of an acceptable scientific standard.

## References

[1] : Pharmacogenetic landscape of Metabolic Syndrome components drug response in Tunisia and comparison with worldwide populations. *PLoS One*.2018;13(4) : 10.1371/journal.pone.0194842 e0194842 10.1371/journal.pone.0194842 29652911PMC5898725

[2] : Association between ABCB1 polymorphisms and response to first-generation antiepileptic drugs in a Tunisian epileptic population. *Int J Neurosci*.2018;128(8) : 10.1080/00207454.2017.1412964 705-714 10.1080/00207454.2017.1412964 29198163

[3] : Cytochrome and sulfotransferase gene variation in north African populations. *Pharmacogenomics*.17(13) : 10.2217/pgs-2016-0016 1415-23 10.2217/pgs-2016-0016 27471773

[4] : High frequency and founder effect of the CYP3A4*20 loss-of-function allele in the Spanish population classifies CYP3A4 as a polymorphic enzyme. *Pharmacogenomics J*.2015;15(3) : 10.1038/tpj.2014.67 288-92 10.1038/tpj.2014.67 25348618
